# The effects of local administration of mesenchymal stem cells on rat corneal allograft rejection

**DOI:** 10.1186/s12886-018-0802-6

**Published:** 2018-06-08

**Authors:** Zhe Jia, Fei Li, Xiaoyu Zeng, Ying Lv, Shaozhen Zhao

**Affiliations:** Tianjin Medical University Eye Hospital, Tianjin Medical University Eye Institute & Tianjin Medical University School of Optometry and Ophthalmology, No. 251, Fukang R., Nankai Dist, Tianjin, China

**Keywords:** Mesenchymal stem cells, Local administration, Cell-based immunomodulatory therapy, Corneal allograft rejection

## Abstract

**Background:**

Mesenchymal stem cells (MSCs) have been reported to promote long-term cellular and organ transplant acceptance due to their immunotherapeutic characteristics. Previous work from our lab using a rat allograft model has shown that systemic infusion of MSCs inhibited corneal allograft rejection and prolonged graft survival. Here, we further investigated the effects of local MSCs administration in the same animal model.

**Methods:**

Donor-derived MSCs were isolated and cultured while corneal grafts obtained from Wistar rats were transplanted into Lewis rat hosts. Hosts were then randomly separated into four groups and treated with previously cultured MSCs at different times and doses. Graft survival was clinically assessed using slit-lamp biomicroscopy and the median survival time (MST) was calculated. Grafts were examined histologically using hematoxylin-eosin (H-E) staining and immunohistochemically using antibodies against CD4. A comprehensive graft analysis of IL-2, IL-4, IL-10, and IFN-γ expression was also conducted using both real-time polymerase chain reaction (PCR) and enzyme-linked immunosorbent assay (ELISA).

**Results:**

Postoperative MSCs injection prolonged graft survival time when compared with controls (MST 9.8 ± 1.2 days). Injection twice of MSCs (MST 12.6 ± 1.4 days) was more effective than a single injection (MST 10.8 ± 1.3 days). MSCs-treated groups also showed suppression of inflammatory cell as well as CD4 + T cell infiltration in the allograft region. IL-4 and IL-10 levels were significantly increased in grafts obtained from postoperative twice MSCs-treated rats when compared with controls. There were no significant differences in IL-2 or IFN-γ expression across groups.

**Conclusions:**

Subconjunctival injection of MSCs in rats was effective in prolonging corneal allograft survival. This effect was mediated by inhibition of inflammatory and immune responses, indicating an anti-inflammatory shift in the balance of T helper (Th)1 to T helper(Th) 2.

**Electronic supplementary material:**

The online version of this article (10.1186/s12886-018-0802-6) contains supplementary material, which is available to authorized users.

## Background

Corneal transplantation is currently the most effective method for visual rehabilitation once deterioration or disease has affected corneal clarity [1]. Although it is also the most successful transplant method, host rejection due to immune responses remains the predominant reason for graft failure [2]. To prevent rejection, current approaches use systemic corticosteroids and immunosuppressants (e.g. cyclosporine A) to prolong corneal graft survival [3]. However, this systemic immunosuppressive approach comes with the attendant risk of drug toxicity and the potential for life-threatening complications. Given this risk, new therapies to ensure the viability of corneal transplantation are in need.

Several studies have demonstrated that mesenchymal stem cells (MSCs) have potent immunomodulatory properties, including immunosuppressive effects that have been shown both in vitro and in vivo [4–6]. Mechanistically, MSCs modulate adaptive immunity by suppressing T cell proliferation and cytokine secretion as well as B cell maturation. In addition, MSCs can also influence innate immunity by inhibiting dendritic cell (DC) maturation and activation as well as natural killer cell (NK) cytotoxicity [7]. In both cases, the governing factors for MSCs’ effects on immunity are through direct cell-to-cell interactions and soluble factor secretion [8].

Given the effects of MSCs on immunity, previous work from our lab sought to understand the effects of MSCs in a rat model of allograft rejection. This work found that MSCs were able to significantly reduce the rate of allograft rejection, with systemic delivery being an effective delivery routine for their administration [9]. The current work presented here sought to improve upon these therapeutic results by extending MSCs efficacy duration and reducing therapeutic dose. We further investigated the effect of local administration of MSCs on corneal allograft rejection in lieu of a systemic approach. Our results indicate that subconjunctival injection of MSCs can prolong corneal allograft survival. Mechanistic results indicated that this effect was due to the inhibition of the inflammatory response and an up-regulation of Th2 cytokines. Taken together, these findings indicate that local MSCs application is a promising, alternative method for the prevention and treatment of immune rejection after corneal transplantation.

## Methods

### Animals

Female Wistar rats (180–220 g) were used to harvest donor corneal grafts. Corneal transplantation was performed on the right eyes of recipient female Lewis (180–220 g) rats. All Wistar and Lewis rats were purchased from the Experimental Animal Center of Academy of Military Medical Sciences (Beijing, China). All animals were maintained at 25 ± 1 °C with relative humidity of 40 ± 5% under 12 h light-dark illumination cycles (8 am to 8 pm). The animals were fed with food and water ad lib.

The experimental protocol was approved by the Ethical Committee of Tianjin Medical University. All animal procedures and protocols were approved by the Laboratory Animal Care and Use Committee of the Tianjin Medical University and handled in accordance with the ARVO Statement for the Use of Animals in Ophthalmic and Vision Research.

### Corneal transplantation animal model

Donor and recipient rats were anesthetized with chloral hydrate (intraperitoneal, i.p., 3 mg/kg). Recipient pupils were completely dilated using 0.5% tropicamide. Donor corneas were obtained from the central corneal region (3.5 mm diameter) using a 3.5 mm trephine and recipient corneal graft beds were simultaneously readied by making a 3 mm diameter button. Donor corneal grafts were then secured onto recipient beds with eight, interrupted 10–0 nylon sutures.

### Mesenchymal stem cell (MSCs) preparation

Wistar rats were used for all MSCs derivation and were isolated and maintained as previously described [10]. Briefly, primary MSCs were cultivated in flasks with complete culture medium consisting of DMEM/F12 (Gibco, New York) supplemented with 10% fetal calf serum (FCS, Gibco, New York), 1% L-glutamine (Gibco, New York), 100 U/ml penicillin (Gibco, New York), and 50 mg/ml streptomycin (Gibco, New York). Cultures were maintained at 37 °C in 5% CO_2_ and the medium was changed every three days. When cultures reached 80–90% confluence, adherent cells were harvested and re-plated in new flasks. MSCs were subsequently collected and characterized as to their differentiation in vitro (Additional file 1: Figure S1) under the appropriate culture conditions. Later expression analysis revealed that MSCs were positive for CD90, Sca-1, CD73, and CD44 expression, but negative for CD45, CD34, and CD11b. MSCs from passages 3–5 were used for all later experiments.

### MSCs administration

Recipient rats were randomly divided into one of four groups. Groups B-D were all administered subconjunctival injections (100 μl) 2 × 10^6^ MSCs in phosphate buffered saline (PBS). Group B subjects received one injection before transplantation (day − 3), Group C immediately after transplantation (day 0), and Group D received two injections (a) immediately after transplantation and (b) three days post-op (days 0 and 3). The dose was selected based on previously published work [11], which has been proved to be safety (Additional file 2: Figure S2). Group A subjects were controls and were administered postoperatively with the same volume of PBS.

Preparation of subconjunctival MSCs administration of 0.1 ml PBS containing 2 × 10^6^ MSCs. MSCs from passages 3–5 were collected and suspected in 1 ml phosphate phosphate buffered saline (PBS). Counted the cell numbers and diluted with PBS. The diluted MSCs were used for later experiments. GFP (green fluorescent protein) labeled MSCs (Cyagen Biosciences Co. Ltd., Suzhou, China) were used for tracking experiments.

### Clinical assessment

Clinical evaluations of all grafts were performed using a slit lamp. They were scored daily for corneal transplant rejection for three weeks post-op. Graft rejection was defined according to the criteria presented in Larkin [12]. Specifically, rejection was determined based on the day when graft opacity, edema, and vascularization was moderate to severe as defined by an opacity score ≥ 3 and a total rejection score ≥ 5. Any subject with surgical complications was excluded from the study and replaced with a new recipient rat.

### Histopathological and immunohistochemistry staining

Histopathological evaluation was conducted on day 10 post-transplantation. Briefly, three rats were randomly selected from Groups A and D, euthanized, and their eyeballs removed for evaluation. Tissue was fixed in 10% neutral formalin for 24 h under room temperature, paraffin-embedded, and then sliced into 4 μm sections. Paraffin sections were then stained using a standard hematoxylin&eosin (H&E) protocol [13].

For immunohistochemical assessment, corneal sections were selected and incubated for 30 min with rabbit anti-rat CD4+ polyclonal antibody (Santa Cruz, USA) or CD68+ polyclonal antibody (Santa Cruz, USA). Slices were then incubated with a biotinylated rabbit anti-goat immunoglobulin (ZSGB-Bio, Beijing, China). Slices were rinsed and allowed to react with horseradish peroxidase (HRP) (ZSGB-Bio) at room temperature for 30 min. All slices were counterstained with hematoxylin.

We use ImageJ software (available in the public domain at http://rsb.info.nih.gov) semi-automatically for quantification of positive cells from immunohistochemistry section. Two independent observers who were masked to the conditions of this study counted the staining. The stained sections were pictured under the bright field by a BX51 microscope (Olympus Optical Co. Ltd., Tokyo, Japan). 3 sections from the comparable positions of cornea were selected and the stained cells were counted.

### MSCs tracking observation

For immunofluorescence staining, freshly excised eyeballs were snap frozen in Tissue-Tek optimum cutting temperature compound (Sakura Finetechnical, Tokyo, Japan) and frozen sections of 6 um thick were fixed by 4% paraformaldehyde for 15 min, permeabilized with 0.1% Triton X-100 for 5 min and blocked with normal serum for 1 h. The samples were stained with Alexa Fluor 488-conjugated anti-GFP (Life Technologies) overnight at 4 °C and subsequently with fluorescein-conjugated secondary antibodies at room temperature for 1 h. All staining was examined by the cellSens Standard electronic system (Olympus Optical Co. Ltd., Tokyo, Japan) under the fluorescence microscope (BX51, Olympus Optical Co. Ltd., Tokyo, Japan) after counterstaining with 4′,6-Diamidino-2-Phenylindole (DAPI)(Vectashield; Vector Laboratories, Burlingame, CA, USA).

### Graft Th1 and Th2 cytokine expression

Transcript levels of corneal graft pro- (IFN-γ and IL-2) and anti-inflammatory cytokines (IL-4 and IL-10) were assessed with real-time polymerase chain reaction (real-time PCR). On days 7 and 10 post-op, six rats were euthanized and one cornea from each was harvested. Two corneas collected from two rats in the same group were pooling together as one sample. The collected corneas were thoroughly digested with proteinase K, and total RNA was extracted using TRIzol method according to manufacturer’s instruction. And cDNA was subsequently generated in a 20 μl reaction volume using commercially available reverse transcription PCR (RT-PCR) reagents (Thermo, USA). Generated cDNA was then used with inflammatory cytokine primers to assess relative levels. Forward and reverse primer sequences are as follows:

IFN-γ: F: 5′ -CACGCCGCGTCTTGGT-3′, R: 5′ -GAGTGTGCCTTGGCAGTAACAG-3′;

IL-2: F: 5′ -GCATGCAGCTCGCATCCT-3′, R: 5′ -TTGAAGTGGGTGCGCTGTT-3′;

IL-4: F: 5′- AGGGTGCTTCGCAAATTTTACT-3′, R: 5′ -CCGAGAACCCCAGACTTGTTC-3′; IL-10: F: 5′- CCCTGGGAGAGAAGCTGAAGA-3′, R: 5′- CACTGCCTTGCTTTTATTCTCACA-3′;

GAPDH: F: 5′ - ACAAGGCTGCCCCGACTAC-3′, R: 5′ -CTCCTGGTATGAAATGGCAAATC-3′.

Forward and reverse primers for GAPDH were used as an internal control (see above). Thermalcycling parameters consisted of the following steps: Denaturation for 2 min at 50 °C and 10 min 95 °C followed by 40 cycles of 15 s at 95 °C and 1 min at 60 °C. For each sample, threshold cycle (CT) value of IL-4 was normalized using the formulaΔCT = CT_IL-4_^_^ CT_GAPDH_. MeanΔCT was determined and relative IL-4 mRNA expression was calculated using the 2^ΔCT^ method. This same approach for relative expression was used to evaluate IL-2, IFN-γ, and IL-10 transcript levels. ^-^

### Enzyme-linked immunosorbent assay (ELISA)

Six rats were selected from group A and D and their corneas were harvested. The total protein from each corneal graft was harvested using the commercially available Tissue Protein Extraction Kit (CWBIO, Beijing, China) according to the manufacturer’s instructions. Commercially available ELISA kits (R&D Systems, USA) were used to measure IL-4 and IL-10 concentrations.

### Statistical analysis

Penetrating keratoplasty (PKP) clinical scores were assessed with a Kaplan-Meier analysis for survival time. A one-way ANOVA was used to measure transcript levels of IL-2, IFN-γ, IL-10, and IL-4. All data were analyzed using the statistical package SPSS (version 19.0; SPSS, Inc). Data are expressed as mean ± SD and *p* < 0.05 was considered statistically significant.

## Results

### Rat MSCs characterization

MSCs were harvested from rat bone marrow and subsequently purified by their adherence to plastic culture flasks [10]. Analysis revealed that adherent MSCs had a spindle-shaped, fibroblast morphology. MSCs have the potential to differentiate into multiple cell types, including adipocytes and chondrocytes, depending on the media provided. Phenotypic analysis of MSCs using flow cytometry showed that bone marrow MSCs were positive for CD90 and CD29, but lacked expression for CD45 and CD34 [10].

### Corneal grafts survival

**Fig. 1:**
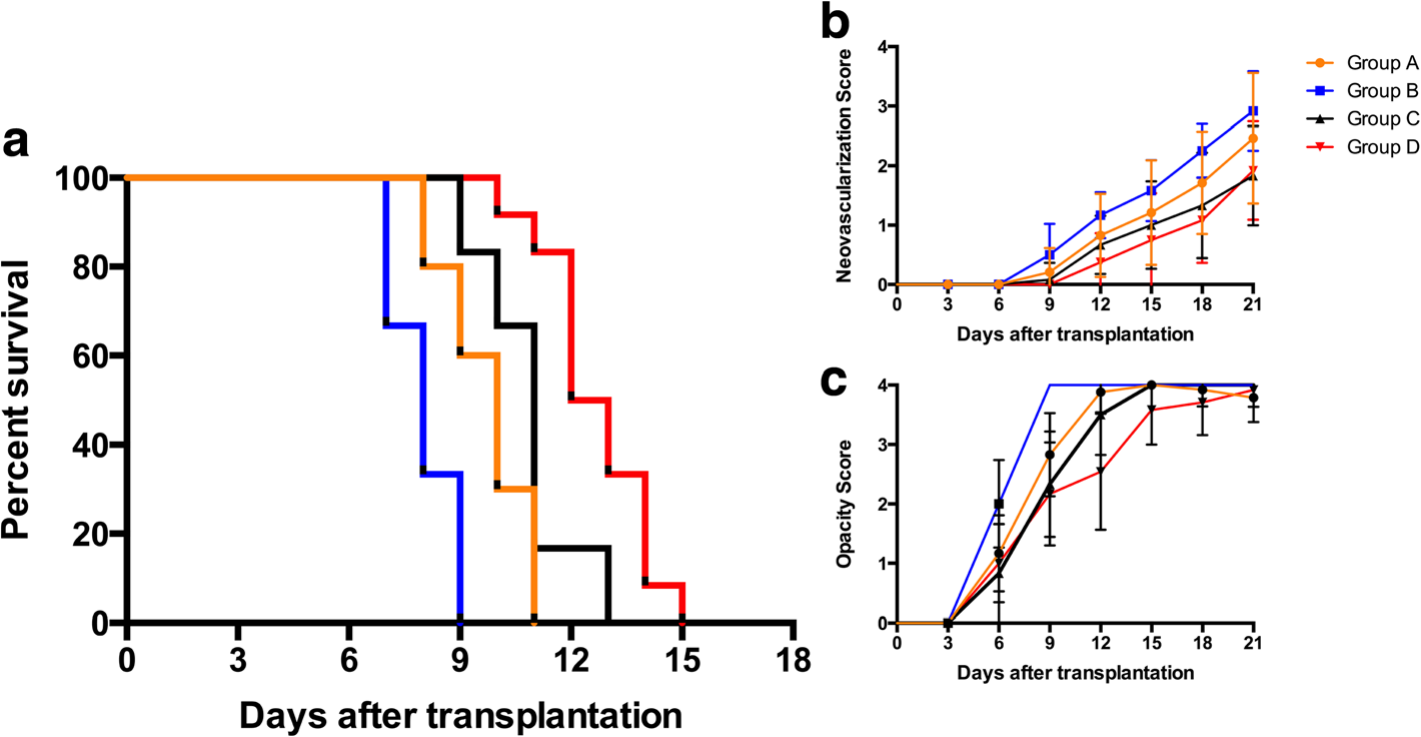
Group survival time The Kaplan–Meier survival plot of corneal allografts in mesenchymal stem cell (MSCs)-treated and untreated rat (**a**). When compared with vehicle-treated rats (Group A) (9.8 ± 1.2 days), rats receiving postoperative, dual injections of MSCs (Group D) (12.6 ± 1.4 days, *p* = 0.002) had significantly prolonged graft survival time. A single MSCs injection (Group C) (10.8 ± 1.3) did not result in statistically significant changes in graft survival time. However, preoperative MSCs (Group B) (8.0 ± 0.9 days, *p* = 0.007) accelerated graft rejection time when compared with controls. The scores of neovascularization (**b**) and opacity (**c**) are shown over time

**Fig. 2:**
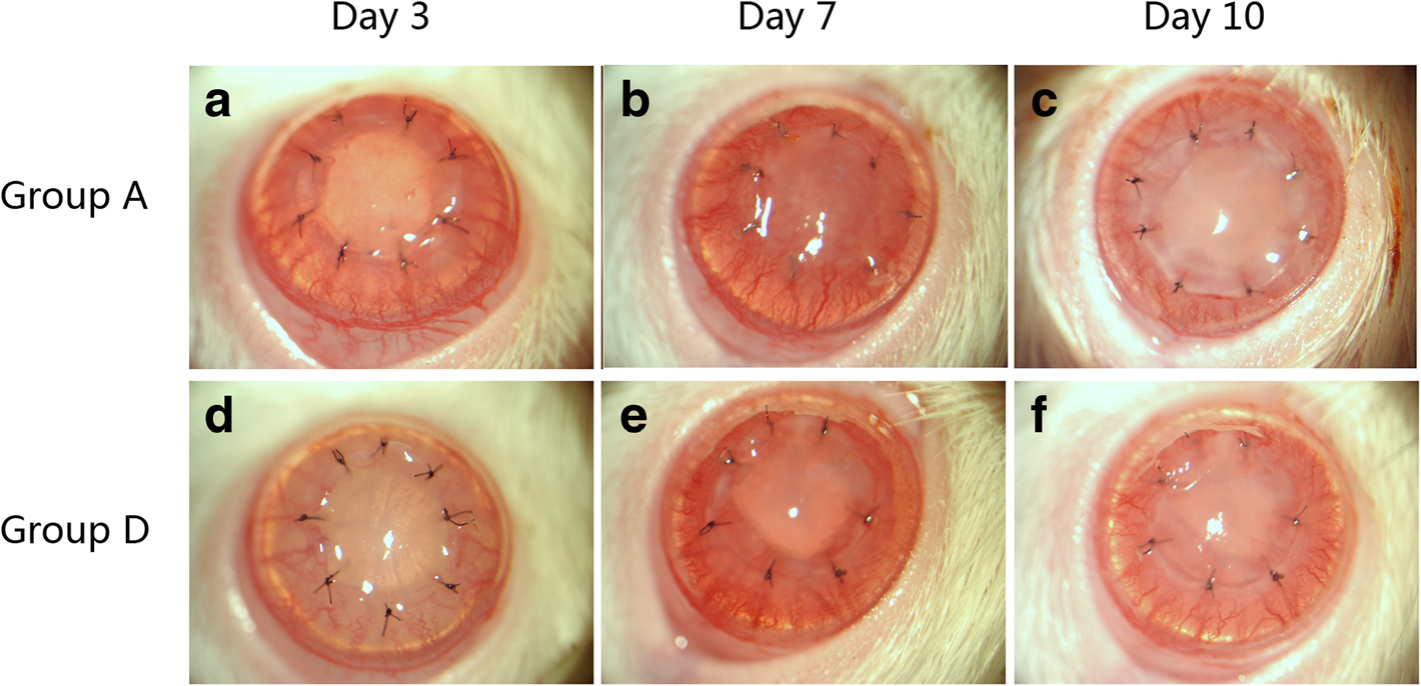
Clinical assessment of grafts. Three days post-op, corneal grafts were transparent in Groups A (**a**) and D (**d**). Corneal neovascularization began to grow into the cornea from the limbus. At day 7 and when compared to controls (**b**), both corneal edema and neovascularization of Group D were mitigated (**e**). By day 10, corneal edema was severe in controls (**c**). The rejected grafts were opaque and a large number of new vessels had grown into the central portion of the grafts. In MSCs-treated groups (**f**), the cornea was still transparent with a pupil and new vessels were not near the peripheral portion of the grafts

To understand the effect of MSCs on corneal transplantation, graft survival in each group was assessed (Fig. 1) and compared. In the control group (Group A), mean graft survival time (MST) was 9.8 ± 1.2 days (Fig. 1a, Table 1). Preoperative MSCs therapy (Group B) accelerated immune rejection with a MST of 8.0 ± 0.9 days. This effect was significant (*p* = 0.007) when compared with control allografts. Group C (single post-op MSCs injection) did not significantly prolong graft survival time when compared with controls (10.8 ± 1.3 days, *p* > 0.05). However, Group D (dual post-op MSCs injection) had significantly prolonged graft survival time when compared with control allografts (12.6 ± 1.4 days, *p* = 0.002) (Fig. 2) (Table 1).

**Table 1:** Corneal allograft survival time(d, *x* ± *s*)

Group	Graft survival time (days)	n	Mean ± SD
A	8 × 4,9 × 6,10 × 6,11 × 6,12 × 2	24	9.8 ± 1.2
B	7 × 4,8 × 4,9 × 4	12	8.0 ± 0.9^a^
C	9 × 2,10 × 2,11 × 6,13 × 2	12	10.8 ± 1.3^b^
D	10 × 2,11 × 2,12 × 8,13 × 4,14 × 6,15 × 2	24	12.6 ± 1.4^a^

^a^p < 0.05 vs Group A; ^b^p > 0.05 vs Group A

### MSCs treatment suppresses inflammatory and CD4 + T cell infiltration

**Fig. 3:**
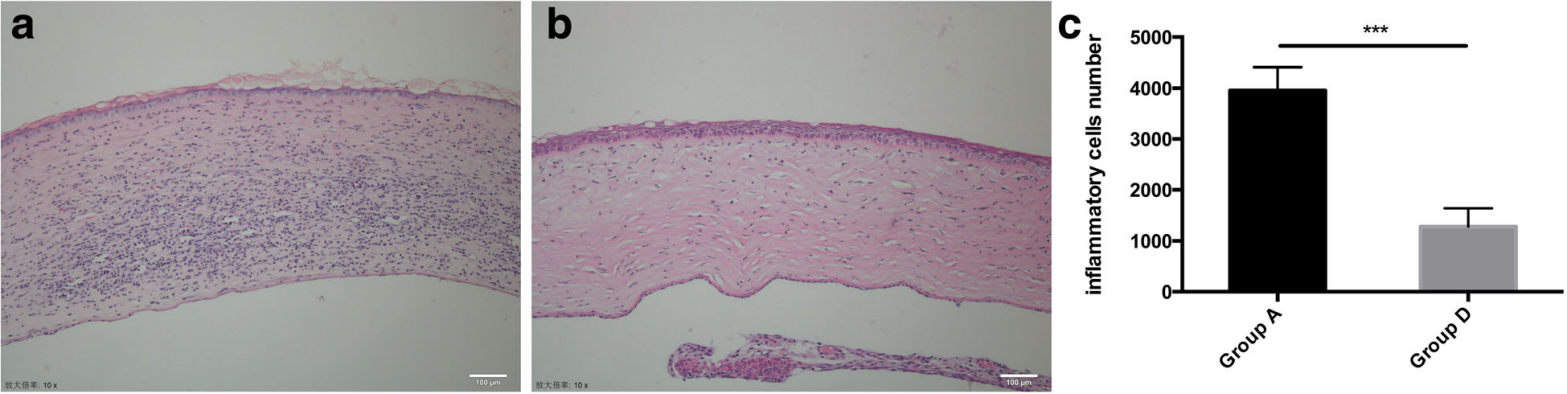
H&E staining. H&E staining in control (Group A) and double injection groups (Group D). H&E corneal graft staining at day 10 showed heavy infiltration of inflammatory cells in the rejected allografts of controls (**a**) and much less inflammatory cell infiltration in the Group D allografts (**b**). ***p < 0.001

**Fig. 4:**
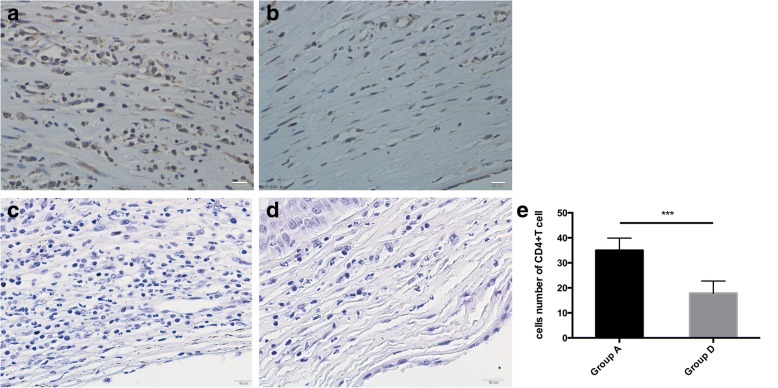
CD4 + T cell immunohistochemical staining. CD4 + T cell immunohistochemical staining in control (Group A) and double injection groups (Group D). Immunohistochemical staining showed that a larger number of CD4+ T cells had infiltrated control group allografts (**a**), whereas there were almost no T cells in MSCs-treated grafts (**b**). No secondary antibody immunohistochemical staining of Group A (**c**) and Group D (**d**). ***p < 0.001

**Fig. 5:**
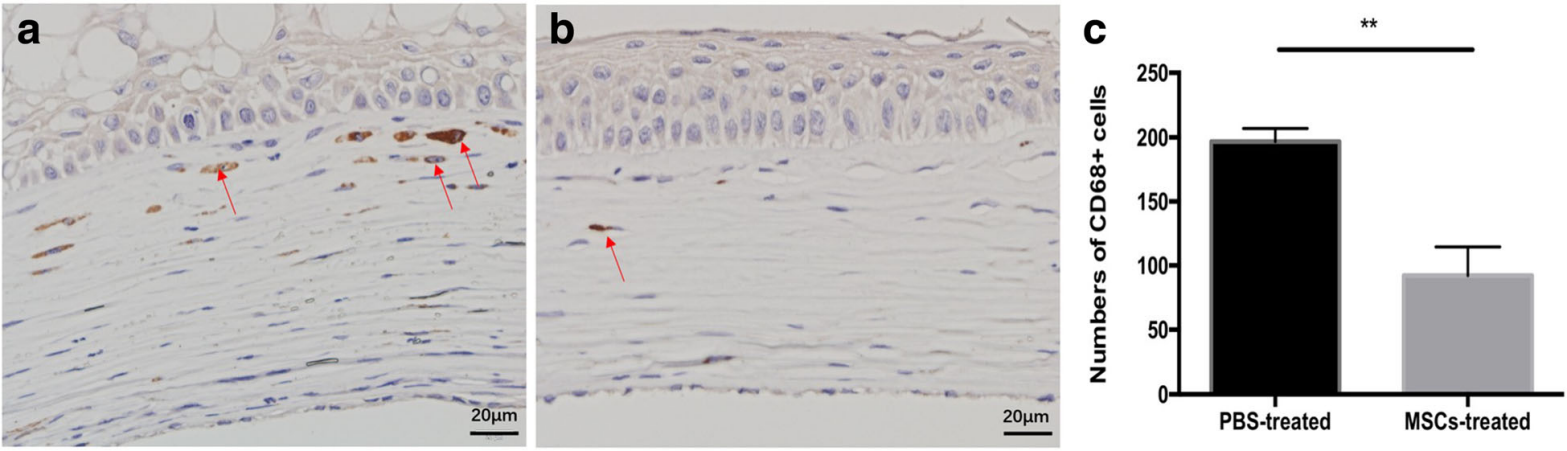
CD68+ macrophages immunohistochemical staining. Immunohistochemical staining of CD68+ marker showed that the number of macrophages cells in grafts of MSCs-treated group (**b**) is obviously decreased than control group (**a**). **p < 0.01

At day 10 after transplantation, corneal allograft rejection was observed in the control group (Group A), Group B, and C; but not in the Group D, which received the MSC subconjunctivally injections on Day 0 and 3. Therefore, the control group without any treatment and group D, the MSC injection group with the optimal effects on allograft survival, were selected for histological analyses at this time point. Epithelial vacuolization and disordered lamellar structure of the stromal collagen were present in the rejected grafts from controls (Group A). H&E staining also revealed extensive infiltration of inflammatory cells (Fig. 3a). In contrast, inflammatory infiltration was markedly decreased in corneal grafts of rats given high MSCs doses (Group D, Fig. 3b). Histologically, such graft had ordered lamellar structure of stromal collagen, indicating reduced inflammation and inhibited rejection. Immunohistochemical results for CD4+ T cells were similar, with extensive infiltration of CD4+ T cells in rejected grafts from Group A (Fig.4a) and only mild infiltration in grafts from Group D (Fig. 4b). Inflammatory cells number (Fig. 3c), CD4 + T cells number (Fig. 4e) as well as CD68+ cell number (Fig. 5) of Group A and Group D were calculated by Image J.

### MSCs effect on Th1/Th2 balance

**Fig. 6:**
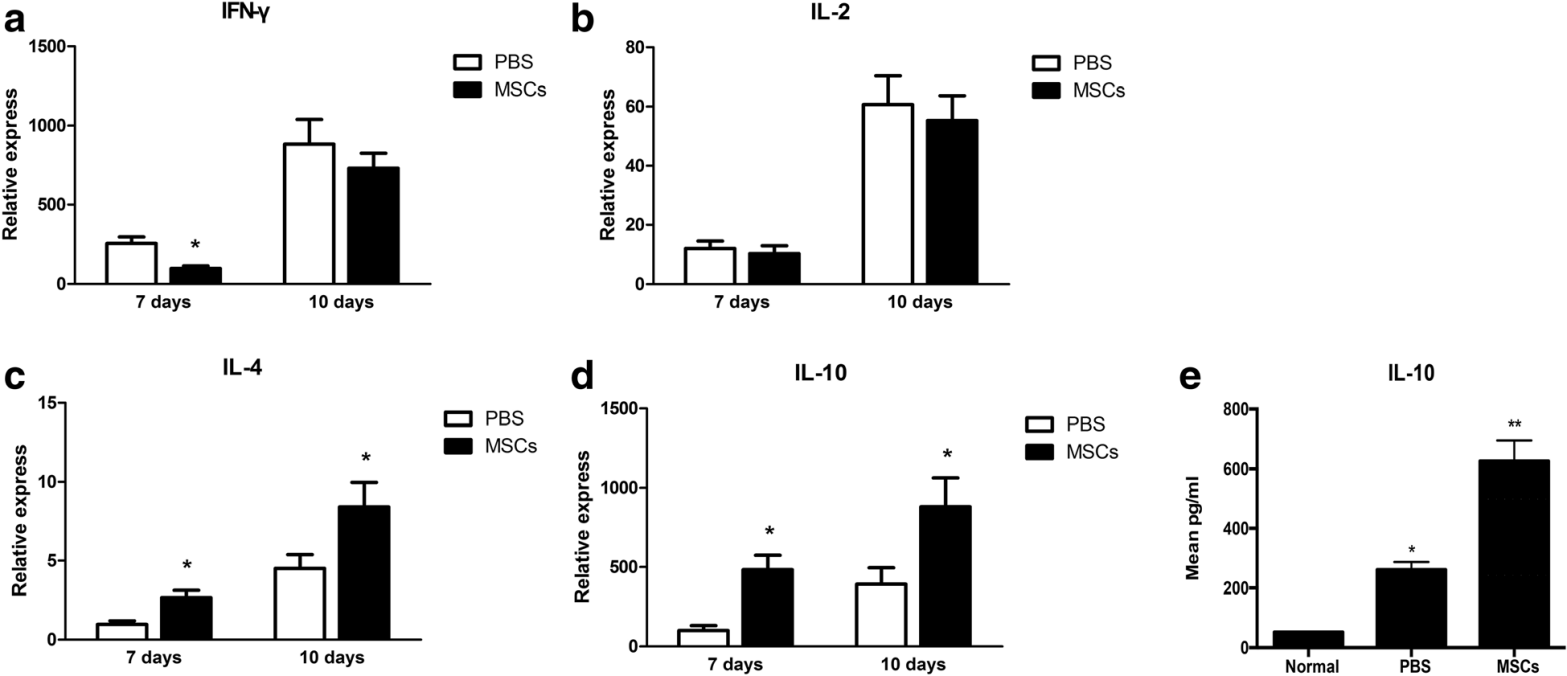
Cytokine expression in corneal allografts. At day 7, IL-4 and IL-10 mRNA expression in MSCs-treated groups was significantly higher than in controls (**c**, **d**). MSCs treatment also reduced IFN-γ mRNA expression (**a**). At day 10, grafts receiving MSCs treatment maintained higher IL-4 and IL-10 mRNA expression (**c**, **d**), but lower IFN-γ and IL-2 mRNA expression (**a**, **b**). Collectively, the Th1/Th2 ratio was reduced in MSCs-treated grafts in this corneal rejection model. Cytokine IL-10 expression was evaluated using ELISA (**e**). At day 10, IL-10 levels were significantly increased in mesenchymal stem cell-treated grafts when compared with untreated grafts and normal cornea (F = 142.92, *p* < 0.05)

To examine the possible mechanisms behind the effectiveness of local MSCs therapy, we next analyzed corneal grafts for the time course of inflammation- and immune-related transcript levels. To this end, IFN-γ, IL-2, IL-10 and IL-4 were examined at days 7 and 10 post-op using quantitative real-time PCR (Fig. 6).

At day 7, clinical scores of grafts were not reached rejection from control group (Group A). However, IL-4 and IL-10 mRNA expression in MSCs twice injection group (Group D) were significantly higher than in controls. MSCs treatment also resulted in reduced IFN-γ mRNA expression (Table 2). As a result, the Th1/Th2 ratio was also significantly reduced. At day 10, mRNA expression levels of IFN-γ, IL-2, IL-4, and IL-10 were all significantly increased. The grafts of MSCs twice treated group showed a trend towards higher IL-4 and IL-10 mRNA expression, but lower IFN-γ and IL-2 mRNA expression. However, these trends were not statistically different (Table 3). The Th1/Th2 ratio remained reduced in MSCs-treated grafts. Taken together, these results suggest that MSCs shift the balance in our corneal allograft rejection model between Th1 and Th2 towards the latter. This is especially true for Th2 cytokines.

**Table 2:** Immune-related cytokine mRNA expression in rat corneal allografts (day 7) $\left(\overline{x}\pm s,{2}^{-\Delta \Delta Ct}\right)$

Group	n	IFN-γ mRNA	IL-2 mRNA	IL-4 mRNA	IL-10 mRNA
Normal	6	0.000	0.80 ± 0.13	0.54 ± 0.16	1.54 ± 0.43
A (PBS)	6	254.71 ± 40.33^ac^	11.95 ± 2.56^bc^	0.97 ± 0.22^ac^	100.37 ± 31.11^a,c^
D (MSCs)	6	98.35 ± 15.91^c^	10.21 ± 2.71^c^	2.67 ± 0.45^c^	283.68 ± 67.40^c^

When compared with group A, ^a^P<0.05, ^b^P>0.05; when compared with control, ^c^P<0.05 (one-way ANOVA, Bonferroni correction)

**Table 3:** Immune cytokine mRNA expression in rat corneal allografts (day 10) $\left(\overline{x}\pm s,{2}^{-\Delta \Delta Ct}\right)$

Group	N	IFN-γ mRNA	IL-2 mRNA	IL-4 mRNA	IL-10 mRNA
Normal	6	0.000	0.80 ± 0.13	0.54 ± 0.16	1.54 ± 0.43
A (PBS)	6	883.33 ± 155.55^b,c^	60.70 ± 9.69^b,c^	4.51 ± 0.87^a,c^	392.14 ± 103.83^a,c^
D (MSCs)	6	730.833 ± 94.51^c^	55.33 ± 8.40^c^	8.41 ± 1.56^c^	880.90 ± 181.68^c^

When compared with group A, ^a^P<0.05,^b^P>0.05; when compared with control, ^c^P<0.05 (one-way ANOVA, Bonferroni-correction)

We next sought to examine whether MSCs could alter Th2 cytokine levels in corneal allografts. ELISA results indicated that both IL-4 and IL-10 were detected in the grafts. On day 10, the level of anti-inflammatory Th2-type cytokine IL-10 was significantly increased in corneal grafts from MSCs-treated groups when compared with controls (*p* = 0.002, Fig. 6e). IL-4 levels were too low in two groups to be detected (data not showed).

### Tracking of MSCs

**Fig. 7:**
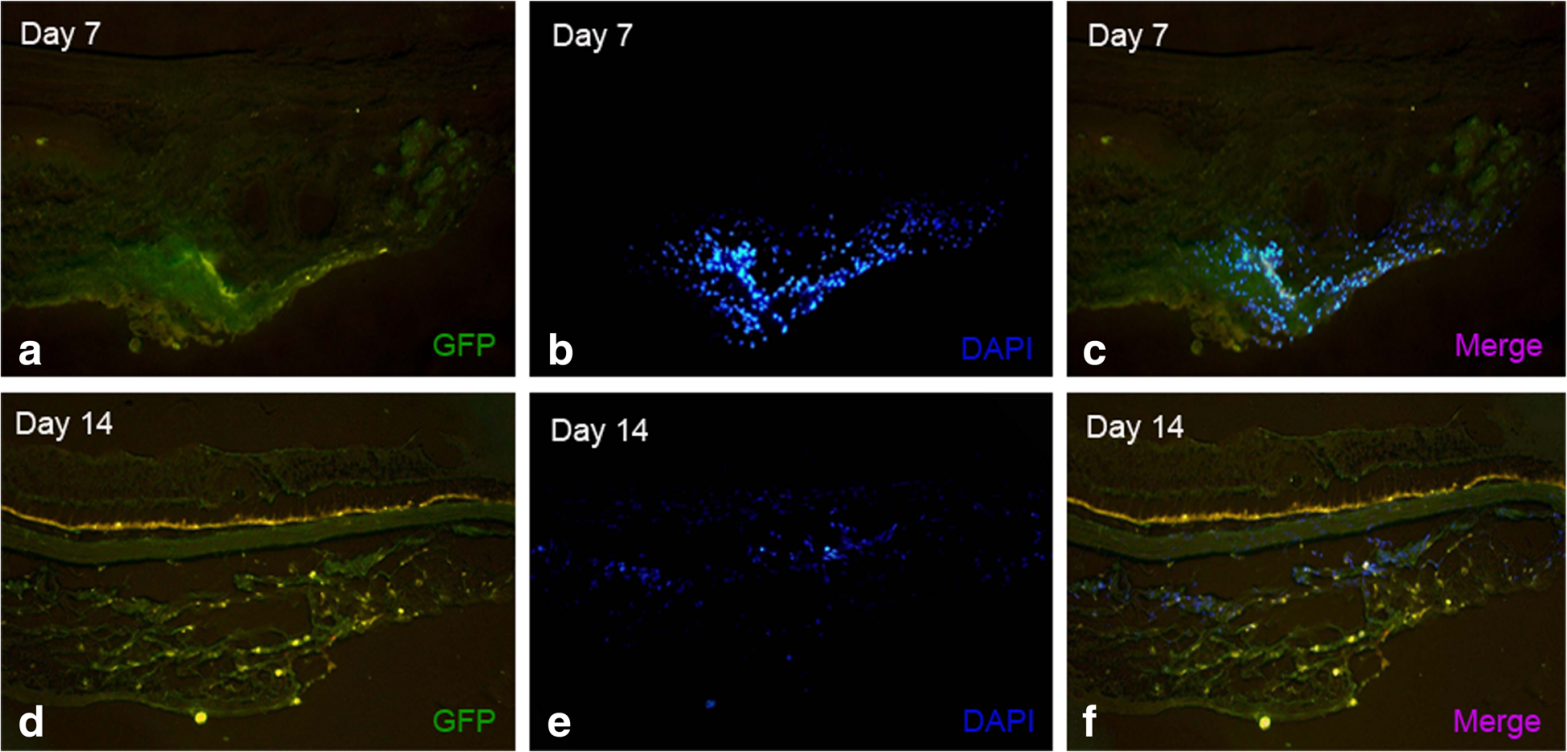
MSCs tracking. GFP and DAPI co-labeled MSCs were used in tracking MSCs in subconjuctival sac. GFP-fluorescence (**a**), DAPI nuclear stain (**b**, **e**) and merged (**c**, **f**) images are shown. After double injection of MSCs (Day 0, Day 3), there is a large quantity of MSCs can be detected on Day 7 (**c**). There are still some MSCs can be checked on Day 14 (**f**)

We use GFP and DAPI co-labeled MSCs to investigate the trace of MSCs after subconjunctival injection. The labeled MSCs were detected via confocal laser scanning microscopy (Fig. 7). After double injection of MSCs (Day0, Day 3), there is a large quantity of MSCs can be detected on Day 7 as well as 7 days after (Day 14). (Operation Day is considered as Day 0).

## Discussion

MSCs have been shown to be effective in a series of studies examining a variety of transplant types. These studies have included both preclinical, experimental work as well as clinical trials. From this work, it became clear that MSCs could effectively modulate immune response. Critically, this could delay immune rejection and prolong graft survival time in heart, kidney, islet, liver, and other organs [14–19]. MSCs have also shown promise in corneal allograft rejection models [9, 20–23]. However, systemic injections of MSCs has obvious disadvantages, including their intrinsic tumorigenic potential and differentiation capabilities [24]. It has also been confirmed that MSCs occur less frequently in sites with tissue damage [22]. After systemic intravenous injection, most cells become trapped in the lungs and other non-target organs, such as the liver, kidney, and spleen [25]. Due to these limitations, local delivery of MSCs has been considered. This would allow MSCs to overcome these biological barriers, thereby modifying their potency, efficiency, and safety [25–28].

The cornea is located on the ocular surface and has a unique immune microenvironment [29]. Local drug administration is a common treatment approach for a variety of ocular surface diseases [30]. To this end, the effectiveness of subconjunctival injection of MSCs has already been validated [11, 31]. For instance, topical MSCs application suppresses inflammation and angiogenesis, in addition to protecting injured corneal cells. Recent studies shows that MSCs provide therapeutic effects through both cell-membrane contact and soluble factors. Local MSCs administration during the acute stage of a rat corneal chemical burn has been shown to facilitate corneal wound repair by the secretion of soluble factors [32]. According to Yao [11], a suspension of 2 × 10^6^ MSCs in 0.1 ml PBS were administrated via subconjunctival injection on days 0 and 3 after a corneal alkali burn. After seven days, a large number of MSCs still remained in subconjunctival sac. However, only few MSCs could found in the wounded cornea tissue. We also speculated that soluble factors secreted by the MSCs rather than cell-membrane contact played a role in subconjuctival injection route. This injection route is thought to improve MSCs concentration as well as soluble factors in the surrounding cornea. We have also shown in the current work that local, subconjunctival MSCs injection is a useful treatment route to delay corneal allograft rejection and prolong corneal graft survival time.

MSCs need to be activated to have an effect. The activation requires an inflammatory microenvironment and stimulation by pro-inflammatory cytokines, either through IFN-γ and TNF-α that are produced from effector T cells or some other connection with immune cells [33]. In previous work, pre-operative injections resulted in longer organ transplant survival times [34, 35]. These studies verified that an intravenous, pre-operative MSCs infusion could modulate regulatory T cell (Treg) expansion early and induce immune tolerance prior to the start of inflammation and the immune response. However, in our study, local MSCs injection prolonged corneal allograft survival only when administered after the operation. Preoperative infusion was shown to accelerate immune rejection. It is possible that preoperative MSCs infusion requires inflammation factors “activated” or “permitted” to play a role in immunological rejection in a microenvironment. Without, they will not effect on immunomodulation. A large number of MSCs that have gathered in the narrow, conjunctival sac might change the microenvironment around the cornea before corneal transplantation. The cornea is an immune privileged tissue and is situated in a special, immune microenvironment that triggers delayed-type hypersensitivity [36]. Although these MSCs did not destroy this status of the cornea, a change of the local corneal microenvironment might be a risk factor for corneal graft rejection. Therefore, as demonstrated here, the postoperative administration of MSCs might be more effective at prolonging graft survival time. Moreover, these results also indicate that MSCs therapy is not always beneficial, and might actually exacerbate disease under certain circumstances.

MSCs dose is another element that influences cell therapeutic effects. During mixed lymphocyte reactions in vitro, MSCs can inhibit T lymphocyte proliferation, but this depends on the graded number of MSCs. In our study, we selected a concentration of suspension of 2 × 10^6^ MSCs in 0.1 ml PBS to inject based on the previous reference which showed to be safe and effective. Our research showed that MSCs administration improved corneal graft survival, but that survival depended on MSCs dose. More specifically, dual injections were more effective than a single injection.

Corneal allograft rejection is mainly mediated by T cells [37] and T helper (Th) cells play the most important role in the immune response. Th1 cells produce pro-inflammatory cytokines IL-2 and IFN-γand are closely associated with graft rejection. Th2 cells secrete IL-4 and IL-10 and can cross-regulate Th1 cytokines, thus contributing to immune tolerance [38, 39]. In general, the balance between Th1 and Th2 is maintained at a relatively stable level, resulting in normal cellular and humoral immune function. In most of transplantation studies, MSCs were able to induce T cell immune tolerance by inhibiting the Th1 response [19, 38]. Here, we found that local application of donor-derived MSCs suppressed the infiltration of inflammatory and CD4+ T cells in grafts. This shifted the Th1/Th2 balance towards a Th2-type response, yielding a significant up-regulation of Th2-response cytokines.

The results presented here suggest that local MSCs injection exerts an immunoregulatory role in corneal transplantation. Moreover, that MSCs-derived therapy is an effective therapeutic strategy to prolong corneal grafts survivial time. However, corneal graft survival time is still not ideal. On the one hand, in vivo MSCs application alone for immune regulation is not sufficient. The application of MSCs combined with a sub-therapeutic dose of an immunosuppressive agent not only exerts a synergistic function in suppressing the immune response [40], but also reduces the side effects caused by large-doses of immunomodulators administered alone. One the other hand, Th1 cells are dominant in graft rejections. Although Th2 cytokines were notably up-regulated in our study, the shift in balance between Th1 and Th2 was not critical enough to prolong corneal allograft survival.

## Conclusions

In conclusion, we demonstrated that subconjunctival MSCs injection suppressed corneal allograft rejection to some extent. Postoperative MSCs injection prolonged graft survival time, with dual MSCs injections being more effective than a single injection. This effect was mediated by inhibition of inflammatory and immune responses, indicated by an anti-inflammatory shift in the Th1/Th2 balance. Although the survival time was not nearly long enough, these findings may offer some value regarding treatment strategies in using MSCs for corneal transplantation.

## Additional files

Additional file 1: **Figure S1.** Differentiation potential of MSCs. (A)Morphology of bone marrow derived mesenchymal stem cells of Wistar rat. (B) Osteogenesis was observed by the formation of the matrix mineralization in Alizarin Red staining. (C) Adipogenesis was observed in MSCs by the formation of lipid droplets with Oil Red O staining. (TIF 8423 kb)

Additional file 2: **Figure S2.** MSCs toxicity test, H&E staining of ocular structure for MSCs toxicity test. Figure series 1–3 represented conjunctiva, cornea and retina, respectively. Series of Figure A are control group and series of Figure B are MSCs-treated group. (JPG 406 kb)

## Abbreviations

DAPI: 4′,6-Diamidino-2-Phenylindole
DCs: Dendritic cells
ELISA: Enzyme-linked Immunosorbent Assay
FCS: Fetal calf serum
GFP: Green fluorescent protein
H&E: Hematoxylin&eosin
MSCs: Mesenchymal stem cells
MST: Median survival time
NKs: Natural killer cells
PCR: Polymerase chain reaction
PKP: Penetrating keratoplasty
