# Supplementary figures and images for: The effects of local administration of mesenchymal stem cells on rat corneal allograft rejection
Source: BMC Ophthalmol. 2018 Jun 8;18:139. doi: 10.1186/s12886-018-0802-6 (PMC5994063; doi:10.1186/s12886-018-0802-6)

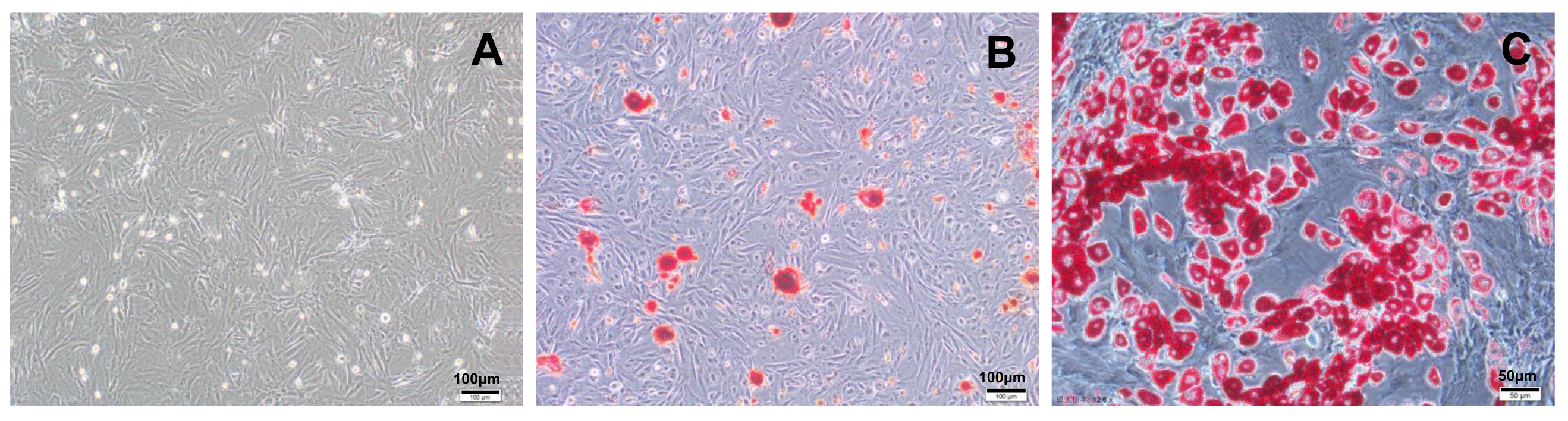

Supplement: Supplementary file 1 — Figure S1. Differentiation potential of MSCs. (A)Morphology of bone marrow derived mesenchymal stem cells of Wistar rat. (B) Osteogenesis was observed by the formation of the matrix mineralization in Alizarin Red staining. (C) Adipogenesis was observed in MSCs by the formation of lipid droplets with Oil Red O staining. (TIF 8423 kb) [file 12886_2018_802_MOESM1_ESM.tif]

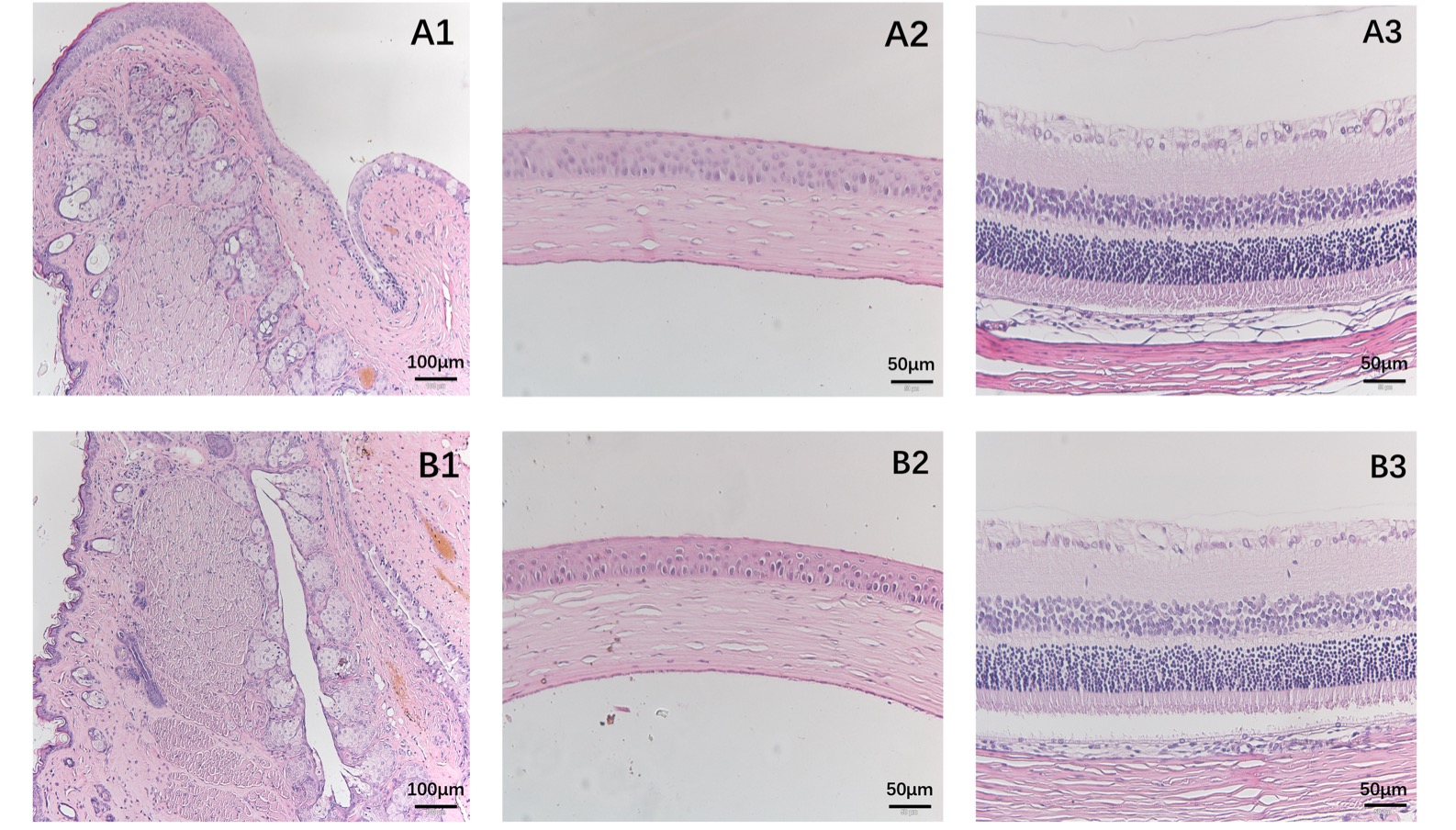

Supplement: Supplementary file 2 — Figure S2. MSCs toxicity test, H&E staining of ocular structure for MSCs toxicity test. Figure series 1–3 represented conjunctiva, cornea and retina, respectively. Series of Figure A are control group and series of Figure B are MSCs-treated group. (JPG 406 kb) [file 12886_2018_802_MOESM2_ESM.jpg]
